# Peritoneal implantation of pheochromocytoma 8 years after adrenalectomy: A rare cause of delayed recurrence

**DOI:** 10.1016/j.radcr.2026.04.059

**Published:** 2026-05-20

**Authors:** Aqeel Alhashim, Abdullatif Alsharif, Hameed Aljawad, Mamdouh Alnahwi

**Affiliations:** a Department of Radiology, Almoosa Health Group, Al Mubarraz, KSA; b Radiology and Nuclear Medicine Department, School of Medicine, University of Jordan, Amman, Jordan; c Department of Pathology and Laboratory Medicine, Almoosa Health Group, Al Mubarraz, KSA; d Department of General Surgery, Almoosa Health Group, Al Mubarraz, KSA

**Keywords:** Pheochromocytoma, Peritoneal implantation, Pheochromocytomatosis, MIBG, Adrenalectomy, Delayed recurrence

## Abstract

Peritoneal implantation of pheochromocytoma following adrenalectomy is an exceptionally rare mechanism of recurrence, typically related to intraoperative tumor spillage or capsular rupture. It may present after a prolonged latency and mimic metastatic disease. We report the case of a 43-year-old man who presented with occasional palpitations, headache, and hypertension 8 years after right adrenalectomy for pheochromocytoma. Imaging revealed multiple peritoneal soft-tissue lesions. I-123 MIBG scintigraphy demonstrated avid uptake in these lesions, confirming peritoneal implantation of pheochromocytoma. This case highlights the importance of considering peritoneal implantation even years after surgery and the value of functional imaging in diagnosis and follow-up.

## Introduction

Pheochromocytomas are rare catecholamine-secreting tumors arising from chromaffin cells of the adrenal medulla. Surgical resection is the treatment of choice and is curative in most cases however, recurrence may occur for multitudes of reasons, including metastatic disease, multifocal tumors, hereditary syndromes, incomplete resection, or iatrogenic tumor cell implantation following capsular rupture or tumor spillage during surgery. Peritoneal or retroperitoneal implantation of pheochromocytoma—occasionally referred to as pheochromocytomatosis—has been described only in a handful of case reports and small series. The cases are characterized by multifocal nodules developing in or around the surgical bed after a prolonged remission interval. We present a radiologic case report of delayed peritoneal implantation of pheochromocytoma diagnosed 8 years after adrenalectomy, emphasizing on imaging findings and clinical outcome.

## Case presentation

A 43-year-old man with no known comorbidities presented to the emergency department with a 2-week history of severe sharp right flank pain, with no aggravating or relieving factors, associated systemic or urinary symptoms. His past surgical history was significant for a right adrenalectomy performed in 2015 in the United States for pheochromocytoma. Details regarding intraoperative tumor handling were unavailable. The patient had remained asymptomatic for several years following surgery and was under regular surveillance with biochemical testing and imaging, all of which had been unremarkable. He had no history of chemotherapy or radiotherapy.

On presentation, vital signs were within normal limits except for elevated blood pressure (170/87 mmHg).

**Fig. 1:**
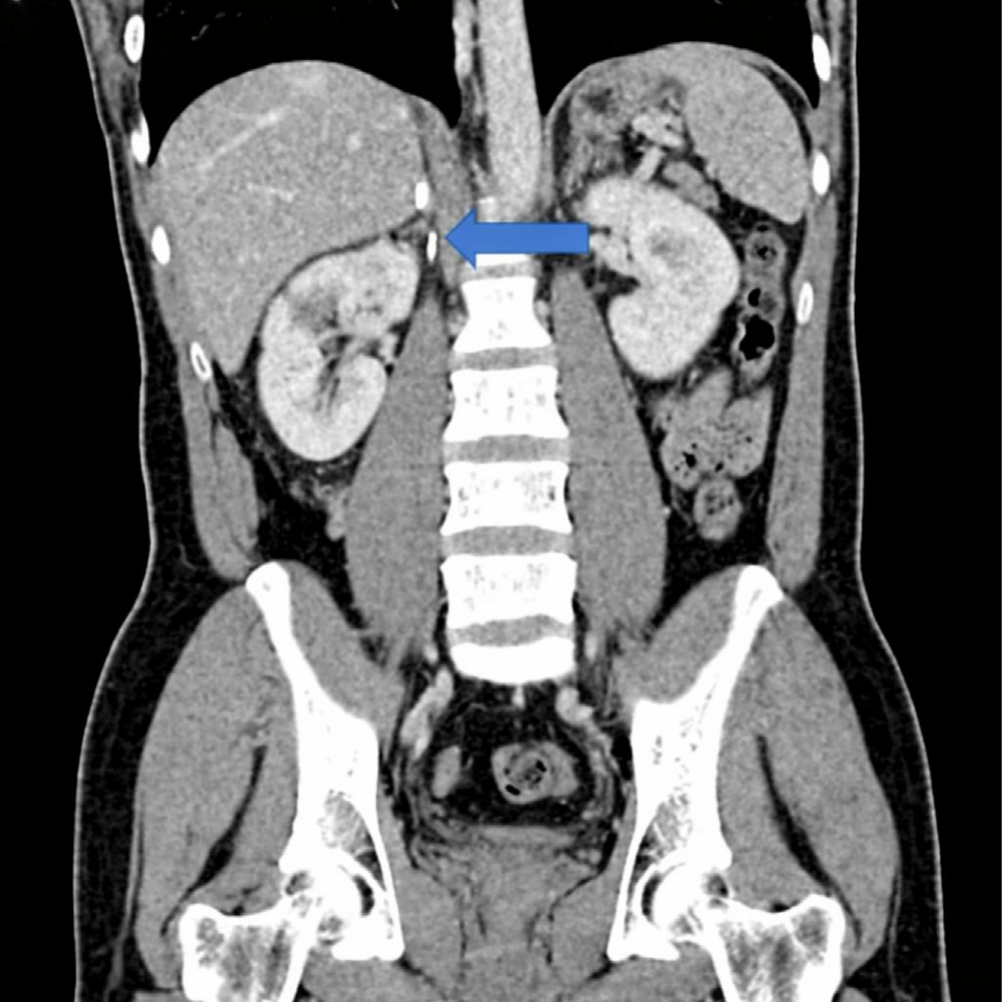
Contract enhanced CT showing surgical clips in the right adrenalectomy bed, with no soft tissue lesions around it.

**Fig. 2:**
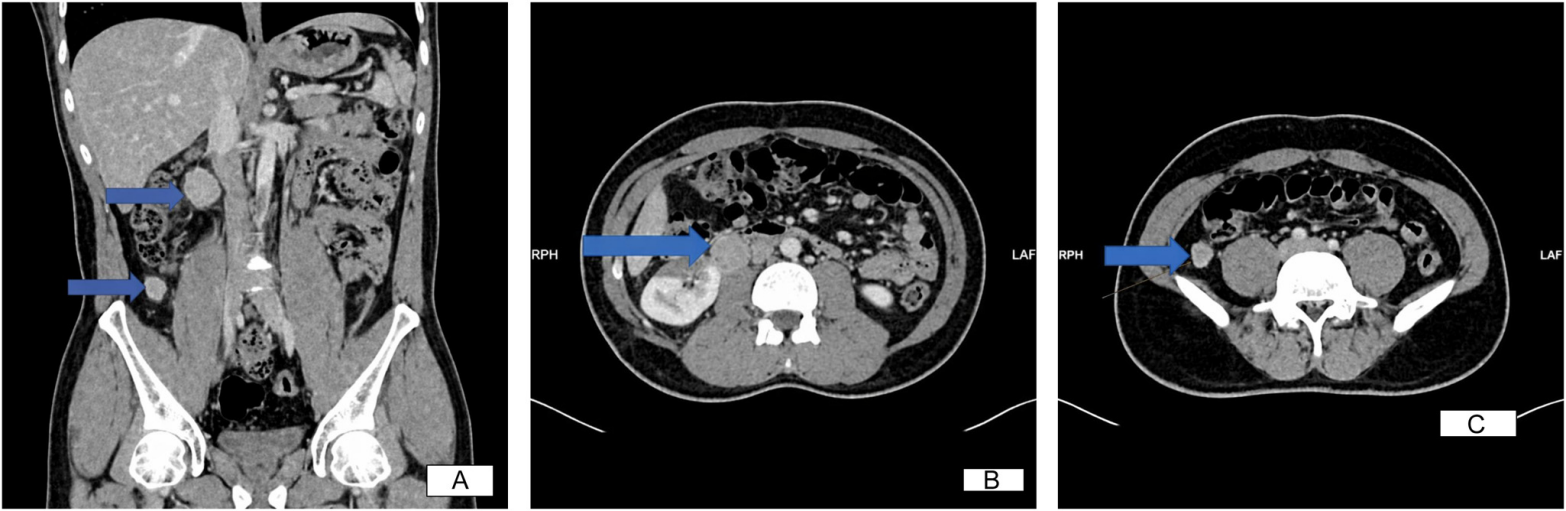
(A-C) – Six well–defined retroperitoneal hypodense homogenously attenuating soft tissue lesions in the renal hilum measuring 2.8 × 25 × 3 cm, and clustered in the right paracolic gutter, and abutting the right psoas muscle with smooth margin and no evasion or encasement of the adjacent organs. The lesions in the paracolic gutter are averaging 2 × 2 × 2.5 cm. (arrows).

Contrast-enhanced computed tomography (CT) of the abdomen done on January 14, 2023, demonstrated surgical clips in the right suprarenal region (Fig. 1). A wedge-shaped, non-enhancing area was noted in the lower pole of the right kidney, consistent with renal infarction, likely accounting for the patient’s flank pain. Additionally, multiple soft tissue nodules were identified in the right renal hilar region and along the right paracolic gutter adjacent to the ascending colon (Fig. 2). These findings raised concern for metastasis of an unknown primary origin or recurrence of pheochromocytoma, and further functional imaging was recommended.

The patient was admitted for further evaluation. Upon detailed history-taking, he reported intermittent episodes of headache and palpitations over the preceding months. Laboratory investigations revealed markedly elevated catecholamine metabolites, with urinary metanephrine measuring 1177 µg/g creatinine (reference range: 29–158 µg/g creatinine) and normetanephrine measuring 2166 µg/g creatinine (reference range: 34–461 µg/g creatinine).

An 18F- FDG PET/CT performed on January 17, 2023, demonstrated no evidence of hypermetabolic recurrence at the adrenal surgical bed. However, there was a moderately hypermetabolic soft tissue lesion at the right renal hilar level abutting the right psoas muscle measuring approximately 2.6 × 2.7 cm with an SUVmax of 3.9; the finding mostly represented metastatic lymphadenopathy. There was no evidence of other hypermetabolic abdominal or inguinal lymph nodes.

**Fig. 3:**
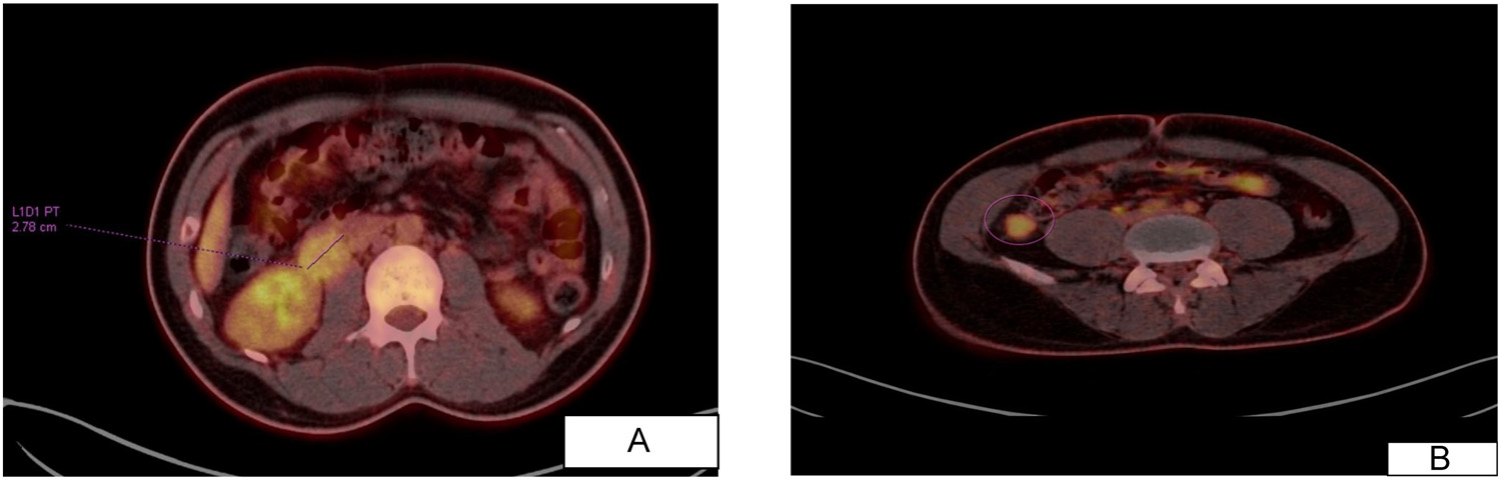
(A, B) – 18F-FDG PET/CT shows no evidence of hypermetabolic disease recurrence at the surgical bed, with a moderately hypermetabolic soft tissue lesion (SUVmax: 3.9) at the right renal hilar level abutting the right psoas muscle. Also, a few mildly to moderately hypermetabolic soft tissue nodules at the right paracolic gutter inferior to the right kidney, some of them abutting the right psoas muscle, the largest measures 1.4 × 1.7 cm with an SUVmax of 3.7.

There were a few mildly to moderately hypermetabolic soft-tissue nodules in the right paracolic gutter, inferior to the right kidney, some of which about the right psoas muscle; the largest measures approximately 1.4 × 1.7 cm (Fig. 3). No additional hypermetabolic lymphadenopathy was identified in the abdomen or inguinal regions.

The patient was subsequently discharged with a plan for further evaluation using I-123 MIBG scintigraphy.

**Fig. 4:**
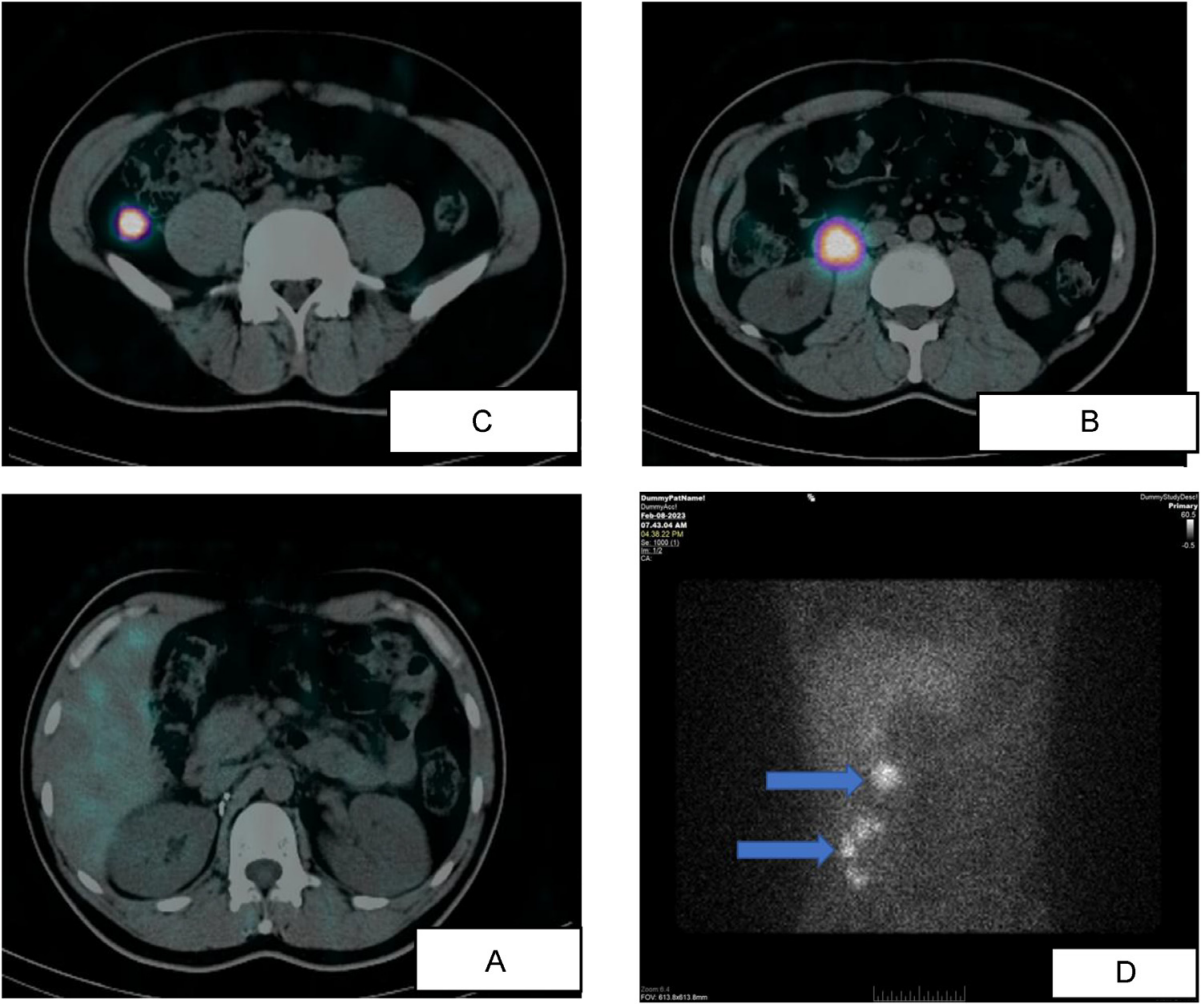
(A–D) – (MIBG) Scintigraphy demonstrating no uptake in the surgical bed, and avid radiotracer uptake in the peritoneal lesions, consistent with catecholamine-producing tissues.

Two weeks later on February 8, 2023, whole-body I-123 MIBG scintigraphy demonstrated avid radiotracer uptake within the corresponding peritoneal lesions, consistent with catecholamine-producing tissue (Fig. 4).

The patient underwent exploratory laparotomy on February 13, 2023. Intraoperatively, multiple peritoneal masses were identified involving the right renal hilum, right hilar lymph node region, right iliac fossa, retroperitoneum, deep retroperitoneum, and lateral retroperitoneum. All lesions were completely excised and submitted for histopathological analysis.

**Fig. 5:**
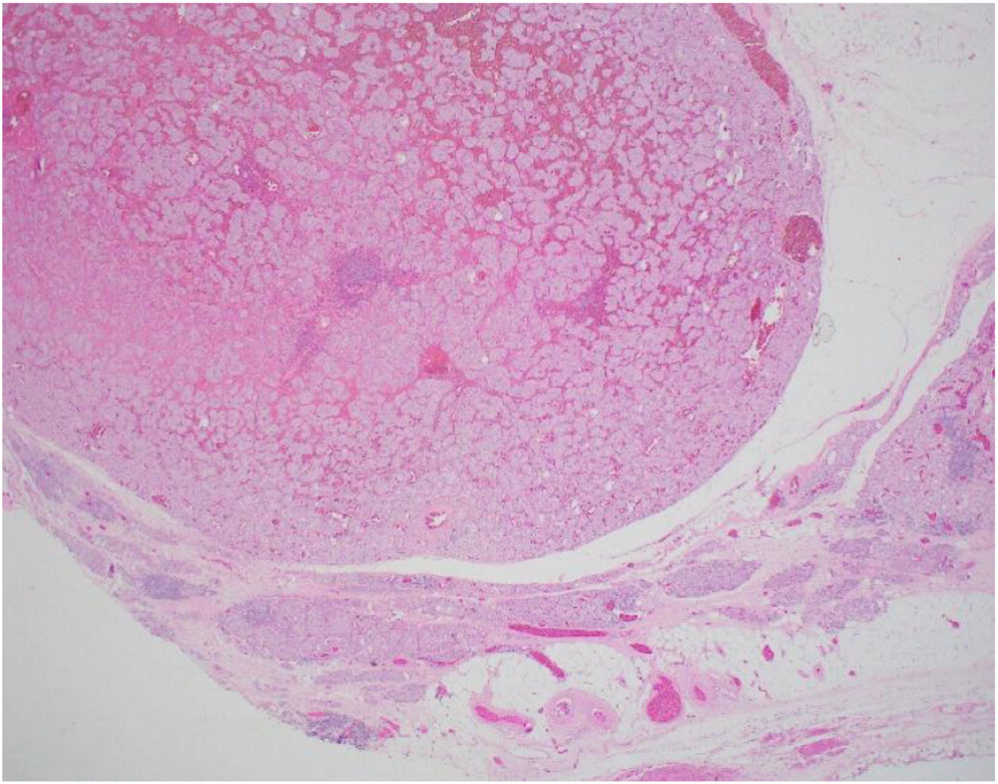
Low power H&E stain (x 2 objective) of retroperitoneal nodule.

**Fig. 6:**
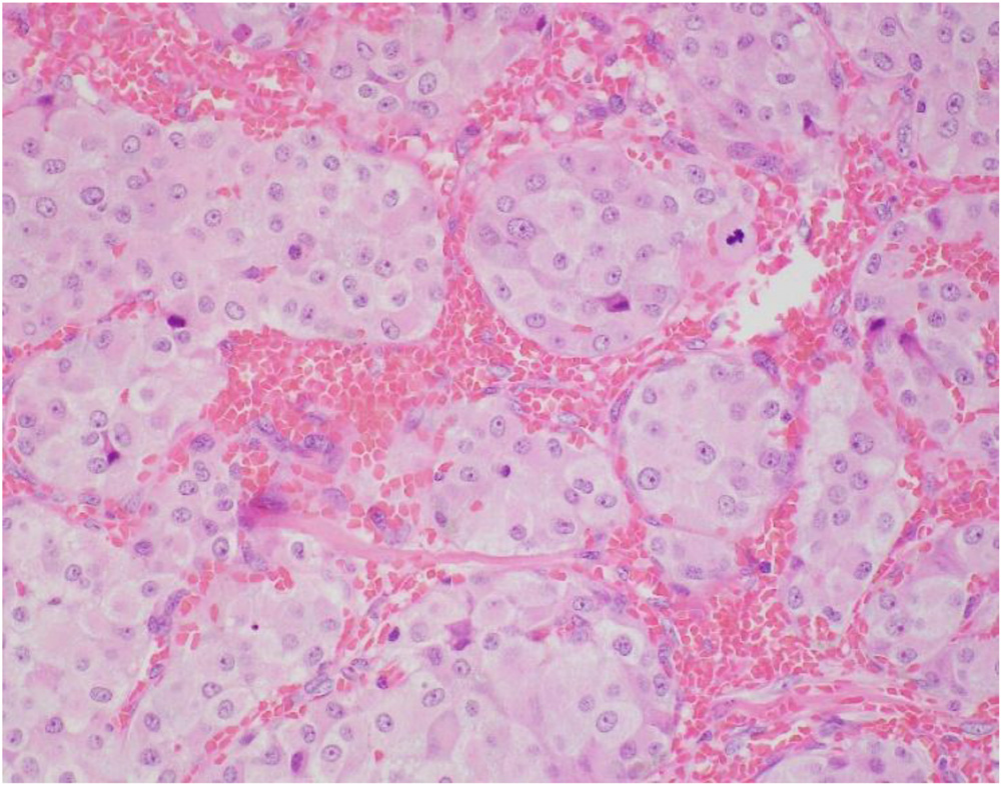
High power H&E stain (x 40 objective) of the tumor nodule cells.

**Fig. 7:**
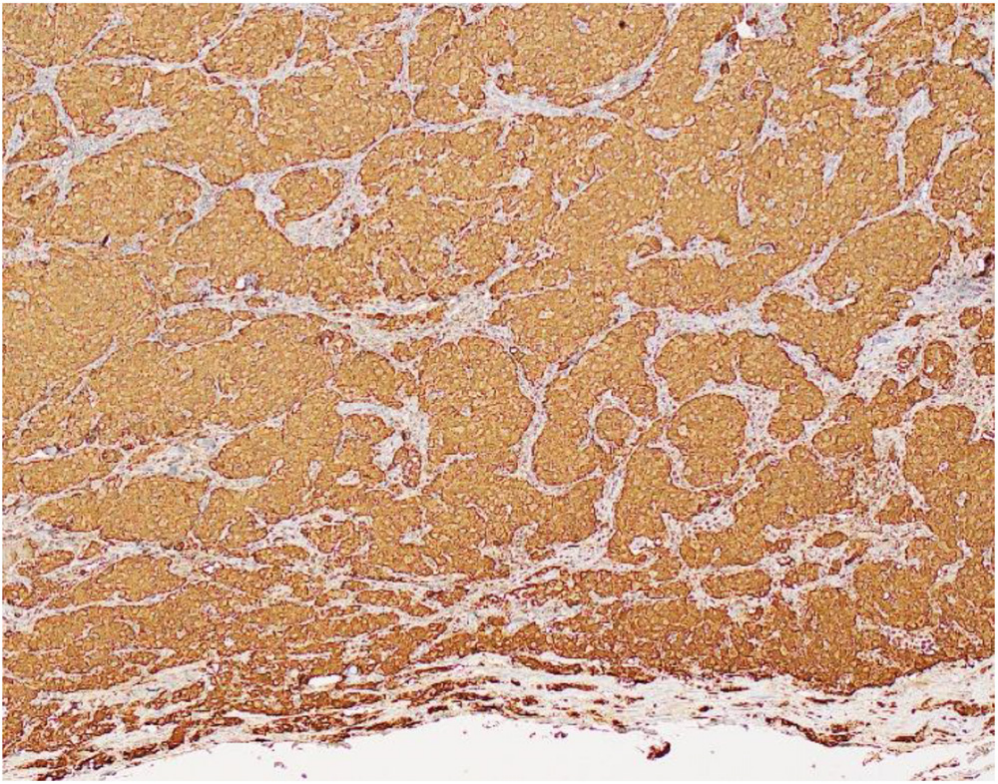
Low power image (x 10 objective), demonstrating strong cytoplasmic staining of tumor cells for chromogranin A immunohistochemistry stain.

**Fig. 8:**
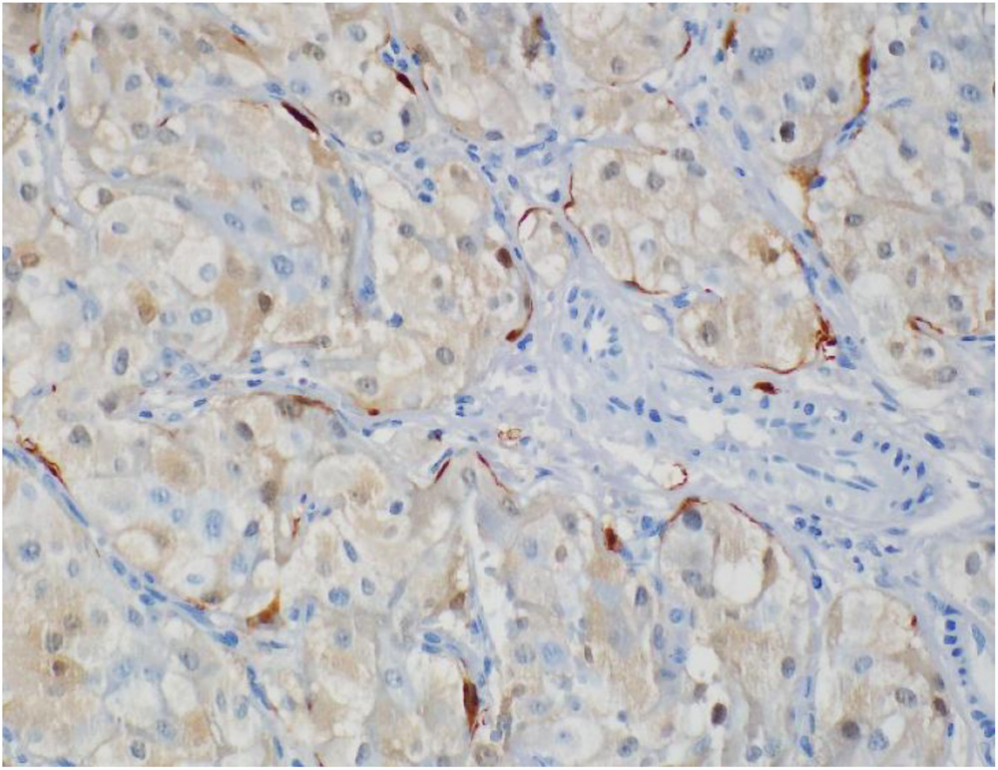
High power image (x 40 objective). S100 protein immunohistochemistry stain highlighted spindle sustentacular cells surrounding some tumor nodules representing zellballen pattern.

Histopathology revealed that all the 6 excised specimens show similar features, each consisting of a single soft tissue nodule. The nodules ranged from 1 to 4 cm in greatest dimension. On sectioning, they were well-circumscribed, unencapsulated, solid, and brown to hemorrhagic, with a surrounding rim of fibroadipose tissue. Low-power examination of a representative retroperitoneal deposit (Fig. 5) shows a well-circumscribed, unencapsulated dominant tumor nodule. At the periphery, particularly in the lower right aspect of the field, there are scattered nests of tumor cells embedded within vascularized fibrofatty tissue. High-power examination (Fig. 6) demonstrates that the tumor is composed of nests (“zellballen” pattern) of uniform polygonal cells with abundant finely granular eosinophilic to amphophilic cytoplasm. The nuclei are round with prominent nucleoli, and mitotic figures are rare. The tumor cell nests are surrounded by spindle-shaped sustentacular cells, highlighting the classic zellballen architecture characteristic of pheochromocytoma. Immunohistochemical studies show strong and diffuse cytoplasmic positivity of the tumor cells for chromogranin A (Fig. 7) and synaptophysin. S100 protein highlights sustentacular cells in a patchy but preserved distribution (Fig. 8). In the context of the provided clinical history and given the absence of residual lymph node architecture in the examined deposits, the histopathologic findings are consistent with pheochromocytoma. However, distinction between metastatic disease and tumor implantation related to prior intraoperative rupture cannot be made on histologic grounds alone and requires clinical and radiologic correlation.

A few days after the surgery, on February 19, 2023, the patient presented to ED with severe diffuse abdominal pain and vomiting. Contract-enhanced CT was done in that episode to exclude obstruction or intra-abdominal collection following surgery.

**Fig. 9:**
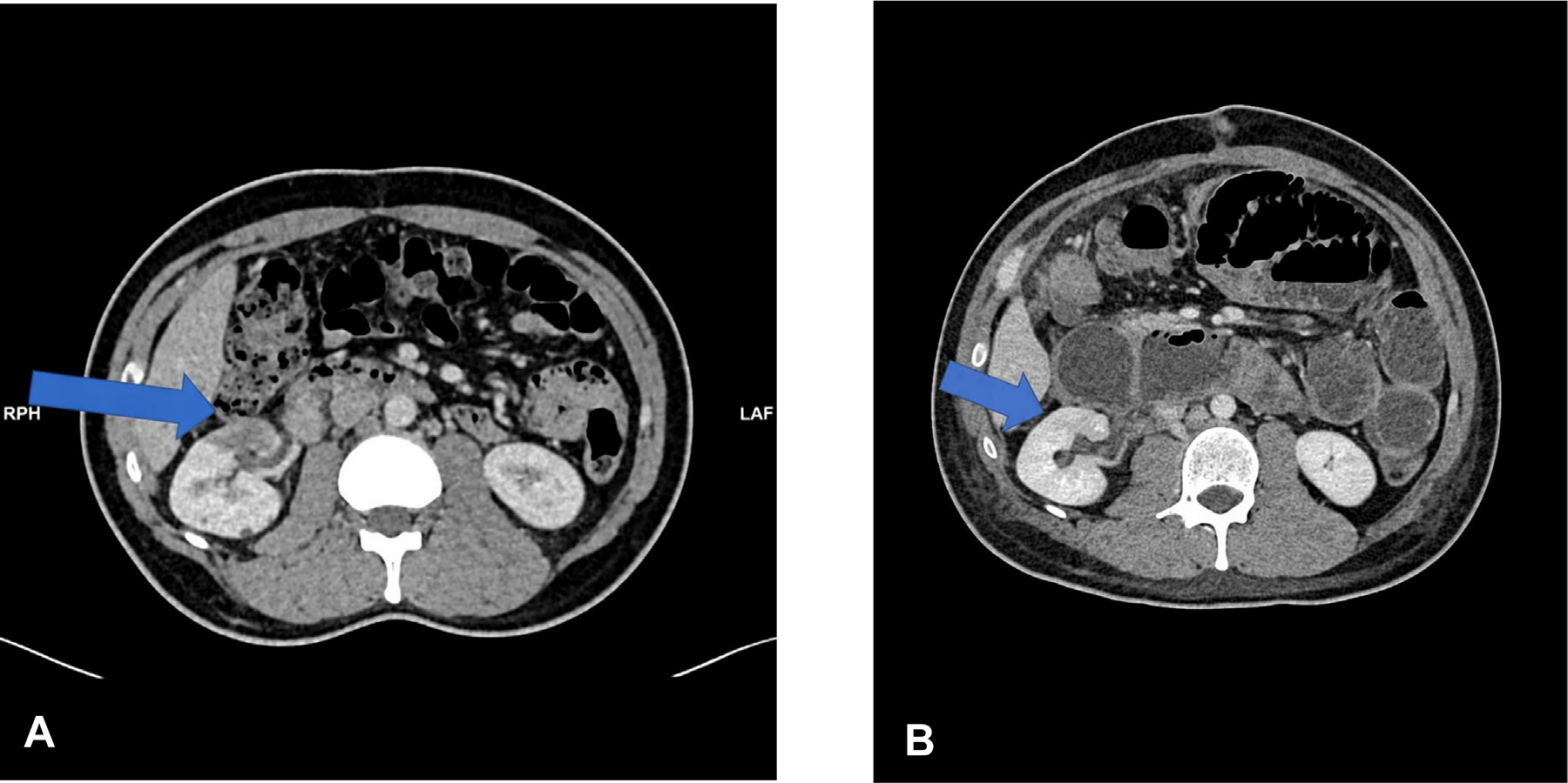
(A, B) – The first CT showing the right renal infarct, and the CT after surgery showing almost complete resolution of the infarct.

The CT showed partial small bowel obstruction and showed significant regression with almost complete resolution of the right renal infarction (Fig. 9). This suggests that catecholamine excess was the etiology of his renal infarction, taking into consideration that the initial workup did not reveal any other etiology for the renal infarct.

Follow-up I-123 MIBG scintigraphy performed several months after surgery on February 2, 2024, demonstrated no residual or recurrent radiotracer-avid lesions. Monthly biological follow-up showed normalization of urinary catecholamine metabolites, with the last one done on March 26, 2025, showing urinary metanephrine measuring 50 µg/g creatinine and normetanephrine measuring 116 µg/g creatinine. The patient remained clinically stable without recurrent symptoms.

## Discussion

Pheochromocytoma recurrence after adrenalectomy is rare but well recognized. While metastatic disease and hereditary predisposition are the most frequently cited causes, implantation of tumor cells within the peritoneal or retroperitoneal cavity represents a rare but increasingly recognized mechanism. Rafat et al. [1] described a series of patients with peritoneal or retroperitoneal implantation of pheochromocytoma, all associated with capsular rupture or tumor fragmentation during initial surgery. These patients experienced long remission intervals before recurrence, and the initial sites of recurrence were consistently regional, supporting direct tumor seeding rather than hematogenous spread.

Pheochromocytomatosis as a term has been used to describe multifocal implantation of pheochromocytoma in the surgical bed or adjacent tissues. Yu et al. [2] reported a classic case following laparoscopic adrenalectomy complicated by capsular rupture, with subsequent development of multiple MIBG-avid nodules. Review of prior cases demonstrated that capsular disruption was a near-universal feature and that recurrence often occurred several years after surgery.

Our case is notable for an exceptionally long latency of 8 years between adrenalectomy and recurrence, exceeding most previously reported intervals. This emphasizes that implanted chromaffin cells may remain dormant for prolonged periods before becoming clinically apparent. Amar et al. [3] highlighted peritoneal inoculation following capsular rupture as a recognized cause of persistent or recurrent catecholamine excess and stressed the importance of lifelong follow-up in patients treated for pheochromocytoma.

Imaging plays a central role in diagnosis. CT typically demonstrates multiple peritoneal or retroperitoneal soft-tissue nodules, which may be indistinguishable from peritoneal metastases of other malignancies. Therefore, functional imaging is critical. 18F- FDG PET/CT has a sensitivity of 85% compared to I-123 MIBG for metastatic pheochromocytoma. However, for nonmetastatic pheochromocytoma, 18F-FDG is of significantly lower sensitivity of 58% while MIBG is of 96% [4]. 8F-FDOPA - which is a radiolabeled amino acid used as a marker of dopamine synthesis for Parkinson’s disease or neuroendocrine tumors – has a much higher sensitivity that could reach to 100 % for benign pheochromocytoma and outperforms I-123 MIBG [5]. However, it is of limited availability and was not readily available in our institution. Thus, I-123 MIBG scintigraphy was pivotal in confirming the diagnosis in this case, as well as in previously reported cases, due to its high sensitivity and it being more readily available. Although PET-based tracers are increasingly used for staging, MIBG remains particularly valuable when confirming tumor origin and assessing suitability for radionuclide therapy.

In our case, the lesions detected in imaging were all in the right paracolic gutter and in the vicinity of the right adrenalectomy bed rather than widespread nodules across the abdominal cavity, which would have been suggestive of metastasis. Additionally, for the whole follow-up period, there was no reported metastasis or recurrence despite the patient not receiving chemotherapy or radiotherapy. Those facts and radiological findings favor peritoneal implantation rather than metastasis from an imaging point of view.

This case also suggests the probability of renal infarction associated with pheochromocytoma due to catecholamine excess, which is a rare phenomenon and reported in a handful of cases [5,6]. However, this is beyond the scope of this case report, and further studies could be done to strengthen this association.

Surgical resection is the cornerstone of treatment for localized implantation disease. While some reported cases required multimodal therapy, including I-123 MIBG treatment, complete surgical excision alone may achieve biochemical and imaging remission when all lesions are resectable, as demonstrated in our patient. Nevertheless, implantation-related pheochromocytoma should be regarded as a potentially malignant condition, and long-term surveillance is essential.

## Conclusion

Peritoneal implantation of pheochromocytoma is a rare yet important cause of delayed recurrence after adrenalectomy and may present decades after initial surgery. Radiologists should consider this diagnosis in patients with a history of pheochromocytoma who develop peritoneal lesions and recurrent catecholamine excess. Functional imaging with I-123 MIBG plays a crucial role in diagnosis and follow-up. Complete surgical resection may result in excellent short-term outcomes; however, lifelong surveillance remains essential.

## Patient consent

Written consent was obtained from the patient for the publication of this article.

## References

[1] Rafat C., Zinzindohoué F., Hernigou A., Hignette C., Favier J., Tenenbaum F. (2014). Peritoneal implantation of pheochromocytoma following tumor capsule rupture during surgery. J Clin Endocrinol Metab.

[2] Yu R., Nissen N.N., Bannykh S.I. (2017). Pheochromocytomatosis after laparoscopic adrenalectomy for pheochromocytoma. Endocr Pract.

[3] Amar L., Fassnacht M., Gimenez-Roqueplo A.P., Januszewicz A., Prejbisz A., Timmers H. (2012). Long-term postoperative follow-up in patients with pheochromocytoma and paraganglioma. Endocr Rev.

[4] Carrasquillo J.A., Chen C.C., Jha A., Ling A., Lin F.I., Pryma D.A. (2021). Imaging of pheochromocytoma and paraganglioma. J Nucl Med.

[5] hewjitcharoen Y., Atikankul T., Sunthornyothin S. (2013). Renal infarction associated with adrenal pheochromocytoma. Urology.

[6] Yang C., Liu K., Huang X., Chen X. (2019). Renal infarction associated with extra-adrenal pheochromocytoma. Urology.

